# Risk Factors of Respiratory Diseases Among Neonates in Neonatal Intensive Care Unit of Qena University Hospital, Egypt

**DOI:** 10.5334/aogh.2739

**Published:** 2020-02-26

**Authors:** Khaled A. Abdel Baseer, Mostafa Mohamed, Eman A. Abd-Elmawgood

**Affiliations:** 1 Department of Pediatrics, Qena Faculty of Medicine, South Valley University, EG; 2 Pediatrics department, Qena university hospital, South Valley University, EG

## Abstract

**Background:**

Respiratory diseases in newborns are considered major causes of neonatal morbidity and mortality especially in developing countries. Its causes are diverse and require early detection and management. This study aimed for detection of the prevalence and risk factors of respiratory diseases in addition to outcome among neonates admitted in neonatal intensive care unit.

**Methods:**

Our study was a prospective observational study that was undertaken at the neonatal intensive care unit of Qena University Hospital, Egypt from July 2017 to July 2018. Demographic and clinical data of newborns and their mothers were evaluated and tabulated.

**Results:**

In this period, 312 neonates were admitted to the neonatal intensive care unit, out of them 145 suffered respiratory diseases giving a prevalence of (46.5%), and (55.9%) were males. The mean neonatal age at admission was 4.33 ± 7.19 days and mean gestational age was 34.49 ± 3.31 weeks. The most common detected respiratory diseases were respiratory distress syndrome (RDS; 49.6%), transient tachypnea of newborn (TTN; 22%), neonatal pneumonia (17.2%) and meconium aspiration syndrome (MAS; 6.21%). Premature rupture of membrane (PROM), maternal diabetes and fetal prematurity had the highest risk factors for respiratory diseases occurrence in neonates. Neonatal mortality rate was 26.2%, mainly due to hyaline membrane disease and pneumonia.

**Conclusion:**

Respiratory diseases constitute major part of total admission in neonatal intensive care unit especially RDS, TTN, pneumonia and MAS. Prematurity and maternal diabetes were the most important risk factors associated with respiratory diseases. Respiratory distress syndrome carried the highest risk of mortality and TTN carried the highest survival rate.

## Introduction

The neonatal period is a very critical period in life due to high possibility of acquiring potential life-threatening diseases and the complexity of the adaptive process of the neonate [1]. According to the American Academy of Pediatrics, approximately 10% of neonates need some assistance to begin breathing at birth, with up to 1% requiring extensive resuscitation [2]. Respiratory diseases are the leading cause of early neonatal morbidity and mortality, as well as the most frequent indication for both term and preterm neonates admission to the special care nursery [3]. In fact, neonates with respiratory distress have 2–4 times more susceptibility to death than neonates without respiratory distress [4]. Neonatal respiratory distress is recognized by one or more signs of increased work of breathing such as tachypnea, chest retractions, nasal flaring and grunting. Normally, the newborn’s respiratory rate is 40 to 60 breaths per minute and tachypnea is defined as a respiratory rate greater than 60 breaths per minute [5]. Certain risk factors increase the susceptibility for neonatal respiratory diseases as prematurity, meconium aspiration, caesarian section delivery, gestational diabetes, maternal chorioamnionitis, and prenatal ultrasonographic findings, such as oligohydramnios and structural lung disorders [6,7]. Regardless of the cause, if not recognized and managed quickly, respiratory disease can progress to respiratory failure and cardiopulmonary arrest. Decreased gestational age predisposes to respiratory distress and there are three times more chances to develop respiratory distress at 37 weeks of gestation than at 39–40 weeks [5]. The underlying causes of neonatal respiratory distress are diverse and does not always lie within the lungs, so, after initial resuscitation and stabilization, it is important to take a detailed history, perform a physical examination, and if needed radiographic and laboratory evaluation to determine a more specific diagnosis and appropriate management [8]. A thorough history may point to risk factors associated with common causes of neonatal respiratory diseases. A detailed physical examination should focus carefully beyond the lungs to identify non-pulmonary causes of neonatal respiratory distress as airway obstruction, cardiovascular and neuromuscular diseases. A tremendous decrease in mortality rate of neonatal respiratory diseases has occurred over the past six decades in developed countries due to renovations in neonatology care that are lacking in developing ones [9].

## Patients and Methods

The current study was a prospective observational study that was done at the neonatology care unit, Qena University hospital from July 2017 and July 2018 to evaluate the prevalence of respiratory diseases among neonates in neonatal care unit. The study included both full-term and preterm neonates with respiratory diseases. Demographic, clinical and laboratory data were evaluated. Out of total 312 neonates admitted to neonatal intensive care unit, 145 neonates were diagnosed with respiratory diseases; 81 males, 61 females and 3 undefined sex. Through interviewed questionnaires to parents and physical examination of neonates, we studied socio-demographic characteristics, antenatal history, intrapartum history, history of prematurity, gestational age, postnatal age, age of onset of the respiratory disease and clinical findings of neonates. Maternal data obtained included age of mother, status of mother according to gravidity, parity, abortion and live Births. Any obstetric complications like ante-partum hemorrhage (APH), premature rupture of membranes (PROM), preeclampsia, eclampsia or diabetes were recorded. Presence of fetal distress, presence of meconium, place and mode of delivery and whether pregnancy was single, twin or triple gestation also were recorded. Clinical examination included general, chest, cardiac and neurological examination. Regarding radiological investigations, chest x-ray, abdominal ultrasound, CT chest and Echocardiography were done in selected cases when indicated. Regarding laboratory investigations, complete blood count with differential, C-reactive protein and blood culture were done in selected cases when indicated. The study was approved by scientific and ethical committee of Qena Faculty of Medicine, South Valley University. An informed consent was obtained from the parents of the newborn.

## Statistical analysis

Analysis of data was evaluated using SPSS version 20. Values that were recorded as mean and standard deviation were compared using Student’s t test. Differences in proportions were compared by applying the chi-square test. Finally, the univariate linear regression Pearson correlation was performed as indicated. P value <0·05 was considered significant.

## Results

In the period of the study, 312 neonates were admitted to our neonatal intensive care unit; of them 145 cases suffered from respiratory diseases giving an incidence of (46.5%) for neonatal respiratory diseases among total admission. Table 1 showed the socio-demographic data of the studied cases. They were 81 (55.9%) males, 61 (42%) females and 3 (2.1%) undefined sex. The mean neonatal age at admission was 4.33 ± 7.19 days, mean gestational age was 34.49 ± 3.31 weeks, mean maternal age was 27.6 ± 6.8 years and the mean duration of hospitalization at our NICU was 9.37 ± 1.59 days. Regarding the site of the delivery, 76 cases (52.4%) of those born at our hospital needed admission at our neonatal intensive care unit due to respiratory problems, 61 newborns were referred to our unit from other hospitals (42.1%) and 8 referred from private clinic (5.5%). One hundred newborns were from urban areas and 45 from rural areas. Most of cases with respiratory distress (97.24%) occurred after normal pregnancy and 4 cases (2.76%) only after assisted pregnancy. One hundred and seven newborns (73.79%) were single, 26 (17.9%) were one of twins, 9 (6.21%) were triplets and 3 newborns were one of quadruple (2.06%). The onset of respiratory distress was since birth in 91 cases (62.8%), at first day in 26 cases (17.9%) and after first day in 28 (19.3%) cases.

**Table 1:** Socio-demographic data of the studied cases.

Parameter		Number	Percentage

Sex	Male	81	55.9%
	Female	61	42%
	Undefined	3	2.1%
Socioeconomic	Urban	100	68.97%
	Rural	45	31.03%
Site of delivery	Qena university hospital	76	52.4%
	Private clinic	8	5.5%
	Other hospitals	61	42.1%
Mode of pregnancy	Normal	141	97.24%
	Assisted	4	2.76%
Mode of delivery	Vaginal	34	23.45%
	CS	111	76.55%
Onset of respiratory distress	Since birth	91	62.8%
	At first day	26	17.9%
	After first day	28	19.3%
Gestational age (weeks)	full term weeks	42	29%
	preterms (34–<37)	29	20%
	preterm (32–<34)	12	8.3%
	preterm (30–<32)	45	31%
	preterm (28–<30)	9	6.2%
	preterm (24–<28)	8	5.5%
Neonatal age (in days)	Mean ± SD	4.33 ± 7.19	
	Range	1–26	
Gestational age (in weeks)	Mean ± SD	34.49 ± 3.31	
	Range	27–39	
Birth weight (in grams)	Mean ± SD	2108.19 ± 810.92	
	Range	740–3330	
Maternal age (in years)	Mean ± SD	27.6 ± 6.8	
	Range	18–45	
Length of hospital stay (in days)	Mean ± SD	9.37 ± 1.53	
	Range	1–19	

Table 2 showed that preterms presented with RDS were 72 cases (49.6%), full-terms presented with transient tachypnea of newborn were 32 cases (22.1%), pneumonia caused respiratory distress in 25 cases (17.2%), meconium aspiration syndrome in 9 cases (6.2%), congenital diaphragmatic hernia in 3 cases (2.1%), aspiration pneumonia in two cases (1.4%) and tracheosophegeal fistula in two cases (1.4%).

**Table 2:** Different causes of neonatal respiratory distress.

Type	Frequency	Percentage(%)

Respiratory distress syndrome	72	49.6%
Transient tachypnea of newborn	32	22.1%
Respiratory distress due to pneumonia	25	17.2%
Meconium aspiration syndrome	9	6.1%
Congenital diaphragmatic hernia	3	2.1
Aspiration of milk	2	1.4%
Tracheosophogeal fistula	2	1.4%
Total	145	100%

Table 3 showed risk factors of respiratory diseases occurrence. Regarding maternal illness, PROM was a risk factor in 32 cases (22.07%), antepartum hemorrhage in 30 cases (20.6%), maternal diabetes in 25 cases (17.24%), oligohydraminos in 20 cases (13.79%), preeclampsia in 9 cases (6.20%), placenta previa in 3 cases (2.06%) and cardiac diseases in 3 cases (2.06%). Regarding fetal conditions, prematurity was a risk factor in 103 cases (70.97%), multiple gestations in 38 cases (26.20%), meconium aspiration in 9 cases (6.21%) and abnormal fetal monitoring in 24 cases (16.55%). Prematurity had the highest risk for RDS followed by PROM, maternal diabetes and pre-eclampsia. Cesarean section had the highest risk for TTN followed by gestational diabetes and prematurity. Prematurity had the highest risk for pneumonia followed by PROM, congenital anomalies and prolonged labor. Cesarean section had the highest risk for MAS followed by abnormal fetal monitoring and post maturity.

**Table 3:** Risk factors of respiratory diseases.

Risk factors		Frequency	Percentage(%)

Maternal	PROM	32	22.07
	Antepartum hemorrhage	30	20.6
	Diabetes mellitus	25	17.2
	oligohydramnios	20	13. 8
	preeclampsia	9	6.2
	Placenta Previa	3	2.06
	Cardiac disease	3	2.06
	Polyhydramnios	3	2.06
Fetal	Prematurity	103	70.9
	Multiple gestation	38	26.20
	Meconium aspiration	9	6.21
	Abnormal fetal monitoring	24	16.5
Risk factors with RDS	Preterm labor	52	72.2
	PROM	24	33.3
	Maternal diabetes	14	19.4
	Pre eclampsia	13	18
	Oligohydramnios	4	5.5
Risk factors with TTN	cesarean section	19	59.3
	gestational diabetes	17	53.1
	prematurity	13	40.6
	small for gestational age	8	25
Risk factors with pneumonia	Prematurity	17	68
	PROM	12	48
	Congenital anomalies	8	32
	Prolonged labour	5	20
Risk factors with MAS	cesarean section	6	66.6
	Abnormal fetal monitoring	5	55.5
	post maturity	5	55.5

PROM: Premature rupture of membrane, RDS: respiratory distress syndrome, TTN: transient tachypnea of newborn, MAS: meconium aspiration syndrome.

Table 4 showed the outcome in relation to different causes of respiratory diseases. Statistical significant difference was found between outcome and incidence of different causes of respiratory disorders. Death rate increased significantly as the incidence of RDS, MAS and pneumonia increased. During the period of the study the case fatality rate was 38.9% in newborns with RDS, 33.3% in MAS and 16% in pneumonia. The best outcome was significantly higher with TTN.

**Table 4:** The outcome of the different causes of respiratory diseases.

Type	Patients		Mode of discharge		P-value

	No.	%	Improved	Died	

RDS	72	49.66	44 (61.1%)	28 (38.9)	<0.05
MAS	9	6.21	6 (66.7%)	3 (33.3%)	<0.05
Pneumonia	25	17.2	21 (84%)	4 (16%)	<0.001
TTN	32	22.07	32 (100%)	0 (0%)	<0.001
TOF	2	1.38	1 (50%)	1 (50%)	NS
Aspiration	2	1.38	2 (100%)	0 (0%)	<0.001
CDH	3	2.07	2 (66%)	1 (33%)	<0.001
Total	145	100	107 (73.8%)	38 (26.2%)	<0.001

RDS: respiratory distress syndrome, TTN: transient tachypnea of newborn, MAS: meconium aspiration syndrome CDH: congenital diaphragmatic hernia TOF: tracheoesophaeal fistula, P value <0.05 is significant.

Table 5 showed logistic regression analysis of variables affecting the outcome of neonatal respiratory diseases. Many variables had a great effect on the outcome with an odds ratio >1 as male gender, RDS, CDH, pneumonia, apnea, tachypnea >60 c/min, retraction, cyanosis and irritability. Maternal risk factors that had a great effect on the outcome were diabetes, pre-eclampsia, oligohydramnios and overcrowding condition with odds ratio >1. Use of mechanical ventilation, C.S delivery and small for gestational age had an odds ratio >1.

**Table 5:** Logistic regression analysis of variables affecting the outcome of neonatal respiratory diseases.

Variables		Odds ratio	CI 95%		P-value

			Min.	Max.	

Sex	Male	1.698	0.840	2.956	0.042
	Female	0.912	0.904	3.245	0.4
Causes of neonatal respiratory diseases	RDS	1.698	0.840	2.956	0.042
	TTN	1.254	0.751	3.717	0.049
	MAS	1.478	0.892	2.532	0.064
	Pneumonia	1.254	0.751	3.717	0.049
	Aspiration of milk	1.075	0.649	4.128	0.066
	CDH	0.912	0.904	3.245	0.097
	TOF	1.211	0.413	1.716	0.067
Signs of RD	Apnea	7.236	2.003	16.536	0.0021
	Tachypnea >60 c/min	1.137	0.462	3.375	0.764
	Retraction	1.743	0.923	1.978	0.0079
	Abdominal distension	0.738	0.677	1.016	0.758
	Cyanosis	4.235	1.251	9.33	0.0046
Maternal diseases	Diabetes mellites	1.310	0.444	1.965	0.758
	Preeclampsia	1.273	0.581	2.725	0.842
	Placenta Previa	0.643	0.734	1.398	0.529
	Cardiac diseases	0.834	0.619	2.065	0.758
	oligohydramnios	1.056	0.861	1.301	0.523
	polyhydramnios	0.856	0.309	1.478	0.107
	Antepartum hemorrhage	1.623	0.847	2.981	0.0249
	Overcrowding	1.672	0.371	1.213	0.36
Management	Mechanical ventilation	29.432	8.654	141.043	0.000001
	Oxygenation	1.913	0.916	2.071	0.171
Type of delivery	Vaginal	0.758	0.947	1783	0.0945
	Caesarean	1.435	0.935	2.051	0.031
Duration of hospitalization		0.862	0.636	1.045	0.0367
Weight for gestational age	SGA	2.076	1.840	4.179	0.0021
	LGA	1.276	0.869	1.687	0.0723
	AGA	0.934	0.469	1.379	0.648

OR: odd ratio, CI: confidence interval. RDS: respiratory distress syndrome, TTN: transient tachypnea of newborn, MAS: meconium aspiration syndrome, APH: ante-partum hemorrhage, CDH: congenital diaphragmatic hernia TOF: tracheo- esophaeal fistula, SGA: small gestational age, LGA: large gestational age, AGA: appropriate gestational age, RD: respiratory distress, P value <0.05 is significant.

## Discussion

Respiratory diseases are one of the most common reasons for newborn admission to the neonatal intensive care unit [5]. Fifteen percent of term and 29% of preterm infants admitted to NICU develop significant respiratory morbidity specially in infants born before 34 weeks of gestation [10]. The etiologies of RD in newborn are large and diverse, including TTN, RDS, MAS, pneumonia and other miscellaneous causes [6,7]. As there is paucity of studies regarding the cause of respiratory diseases in our localities, our study was conducted to find out the prevalence and etiology of respiratory diseases among admitted neonates. The total cases admitted to our NICU during one year study were 312 cases; of them 145 cases were admitted due to respiratory diseases giving an incidence of (46.5%). This was relatively comparable to the incidence reported by other studies like Verma et al., (39%) and Tochie et al., (47.5%) [11,12]. The present study showed that neonatal respiratory diseases were higher in males (55.9%) than females (44.1%). This was similar to previous studies [13,14]. It is well known that lung growth and development start in the prenatal period and lung maturation is more advanced in the female fetus. Oral movement starts between the 16th and 26th weeks of gestation, reflecting fetal breathing, and this is considered a critical determinant for the development of the lung [15]. Other fundamental regulators of lung maturation are sex hormones. While testosterone secreted by fetal testes having mainly inhibitory effects and delays the surge of surfactant production, oestrogens produced by the placenta have positive effects on both the production of fetal surfactant and on the alveologenesis during neonatal and pubertal periods [16]. Additionally, our study detected that neonates born by a caesarean section have more incidence of respiratory diseases. This was evident by other studies [17,18,19]. Infants born by caesarean section have a larger residual volume of lung fluid, a smaller residual capacity and consequently secrete less surfactant into the alveolar space while during vaginal delivery, as the chest of the infant is squeezed, part of the fetal lung fluid is removed and the adrenergic stimulation associated with vaginal labor releases surfactant into the airways [20]. Respiratory distress syndrome was detected as the commonest respiratory disease and earlier workers had found similar observations [10,11]. RDS in the current study was detected in (49.66%) followed by TTN (22.07%), pneumonia in 17.2% and MAS in 6.2%. Parkash et al., reported RDS in (20.8%), pneumonia in (22.5%), MAS in (16.7%) and TTN in (11.7%) [21]. The study of Abou-Faddan and Nafisa revealed that RDS is the most common neonatal respiratory disease (45.8%) followed by pneumonia and TTN [22]. Regarding risk factors, our study showed that the most common maternal factors for occurrence of respiratory diseases were PROM (22%), antepartum hemorrhage (20.6%), maternal diabetes (17.24%) and oligohydraminos (13.8%). Some of serious complications associated with PROM include chorioamnionitis, neonatal sepsis and preterm labor leading to neonatal pulmonary hypoplasia and respiratory distress syndrome [23]. Similar to other studies, the most commonly detected fetal risk factor for respiratory disorders was prematurity (70.9%) [17,22]. It is well documented that all forms of respiratory morbidity, including TTN, RDS, pneumonia, and pulmonary hypertension, affect late-preterm infants at a higher rate than infants of more advanced GA [24]. A study by De Luca et al., revealed that more than ten folds increase in respiratory morbidity in infants of 34 weeks’ GA compared with term infants [25]. A retrospective study by Kitsommart et al., revealed significantly worse respiratory outcomes including prevalence of pneumothorax, need for positive pressure therapy, and mechanical ventilation assistance in infants of 34 to 36 weeks’ GA compared with infants of ≥37 weeks’ GA [26]. The current study found that multiple gestation pregnancies was associated with high risk of neonatal respiratory diseases (26.2%). This was in accordance with the study of Ziadeh and Badria which reported that the second twin has a higher risk of respiratory distress compared to single newborns and added that caesarean section carried out before the onset of labor increase this risk [27]. Multifetal gestation brings a high risk of maternal complications during pregnancy and postnatal complications for babies specially when born prematurely. The risk of preterm delivery for triplet pregnancies is approximately 90% while in twin gestations is more than 50%, compared with only 10% incidence of preterm labor in single infants [28]. Regarding RDS, the most common risk factor detected in our study was prematurity (37.7%). By Indian study, researchers found that the incidence rates of RDS ranged from 86% at 24 weeks to less than 1% at 39 weeks and mentioned that RDS should be anticipated in any IDM and preterm delivery [29]. The results of our study demonstrated that PROM was also an important risk factor for neonatal RDS. Intrauterine infection and chorioamnionitis caused by PROM can cause injury to the fetal lungs and alveolar type II cells directly resulting in decreased synthesis and release of surfactant. Furthermore, premature birth can result from PROM [30]. Infants of diabetic mothers are more susceptible to RDS occurrence compared to those of non-diabetic mothers of equivalent gestational age as having disturbed pattern of surfactant synthesis in addition to delayed appearance of phosphatidylglycerol [31]. Thirty-eight (26.2%) of the studied 145 newborns admitted with respiratory diseases died and this result was comparable to previous studies [32,33]. Kumar et al. reported mortality rate of 19% in India with effective surfactant administration and respiratory support while Bajad et al. reported mortality of 22.33% and Abdelrahman et al. reported 36.0% in Sudan [32,33,34]. Also, the causes of death followed the differences in the causes of respiratory diseases where statistically significant difference was detected between the outcome and the incidence of variable causes of respiratory diseases. Higher mortality rate accompanied higher incidence of RDS, MAS, and pneumonia while higher survival rate with increased incidence of TTN. During the period of the study, the case fatality rate was (38.9%) in RDS, (33.3%) in MAS and 16% in pneumonia. In a study by Adebami et al., mortality rate was 46.9% in RDS and 40.0% in MAS while Kumar et al. reported mortality of (57.1%) in RDS, (21.8%) in MAS and (15.6%) in pneumonia [32,35]. Many variables had a great effect on the outcome of our studied cases as had an odds ratio >1 like male gender, RDS, CDH, pneumonia, apnea, tachypnea >60/min, cyanosis and irritability where apnea had the highest odd ratio of clinical signs. In the study of Panda et al. apnea attack was recognized as a predictor of infant mortality and Sathenahalli et al. described apnea as a sign of poor outcome in neonatal respiratory distress [36,37]. Furthermore we found that need for mechanical ventilation had the highest odd ratio. The results of the Sabzehei et al. study showed that the outcomes of the NRD had a significant correlation with apnea and respiratory failure requiring mechanical ventilation [38]. Regarding relation between causes of respiratory diseases and their fatalities, our study revealed that RDS had the highest odd ratio followed by MAS in addition to maternal diabetes, preeclampsia, oligohydramnios, living in overcrowding conditions and small gestational age. Logistic regression analysis showed that premature SGA neonates were at a higher risk of mortality than premature AGA infants were. These findings were consistent with previous studies that showed premature SGA infants more liable to bronchopulmonary disease and chronic lung disease [39,40]. Strength of this research include use of a reasonable sample size for study and the situation of the study being a big referral center with a high rate of neonatal admission. Also, being a prospective study avoids bias and shortage of data in retrospective studies that depends on file records. The most important limitation of this study is that it was a single centered study and frequent missed cases that go for local centers.

## Conclusions

This study indicated higher incidence of respiratory diseases in neonates admitted at NICU most commonly were RDS, TTN, MAS and pneumonia. Prematurity was the most important risk factor associated with respiratory diseases in addition to presence of antepartum hemorrhage, multiple gestation and maternal diabetes. Respiratory distress syndrome carried the highest risk of mortality and TTN carried the most survival rate. Although decreasing the incidence through preventive measures is ideal, early recognition and treatment of the common neonatal respiratory diseases will decrease both short- and long-term complications and related mortality of at-risk infants.

## Abbreviations

AGA: appropriate gestational age

APH: ante-partum hemorrhage

CDH: congenital diaphragmatic hernia

C.S: caesarean section

LGA: large gestational age

MAS: meconium aspiration syndrome

NICU: neonatal intensive care unit

PIH: Pregnancy induced hypertension

PROM: Premature rupture of membrane

RD: respiratory distress

RDS: respiratory distress syndrome

SGA: small gestational age

TOF: tracheoesophaeal fistula

TTN: transient tachypnea of newborn

## Declarations

- The work described has not been published previously
- It is not under consideration for publication elsewhere
- Its publication is approved by all authors
- Journal policies detailed have been reviewed
- Permission for use of copyrighted material from other sources (including the Internet) has been obtained
